# Annotations

**Published:** 1907-06-08

**Authors:** 


### ANNOTATIONS.

### Mobility of the Kidneys.

Dr. Hector Mackenzie's communication on this subject to the Medical Society of London is one to be carefully noted. He recognises an "abnormally movable kidney" as one in which it is possible to get the hand completely above the upper end of the organ, and to palpate it in its whole extent. In addition, he defines the "palpable kidney" as one in which, though a considerable part of the kidney can be felt, it is impossible to displace it so as to get the hand above the upper end. Among 2,801 females of all ages who were examined, there were found 449 cases in which the kidneys were palpable and 515 in which they were movable, while of 1,607 males only 25 cases showed palpable and 11 cases movable kidneys. In only a single instance was a movable kidney present on the left side alone, while in 49 cases both kidneys were movable. There seems great reason to doubt the commonly accepted statement that repeated child-bearing is an influential factor in the production of abnormal mobility of the kidneys. An important practical point is the degree of disturbance which such abnormal mobility produces, and here Dr. Mackenzie's figures show that out of 526 patients with definitely movable kidneys 411 were wholly unaware of the existence of the condition. This view was confirmed by other speakers, who agreed generally with Dr. Mackenzie's conclusions and with the advice that surgical measures are seldom advisable. In face of all this it is startling to hear from Dr. Suckling, of Birmingham, that "the daily interference with the elimination of urine and retention in the prolapsed kidney and ureter causes auto-intoxication leading to insanity and other disorders of the nervous system," and that "the frequency of suicide when dropped kidney exists is remarkable."

### The Practical Applications of Scientific Medicine

In the *Times* of May 23rd may be found a leading article dealing in an appreciative spirit with the advances which have distinguished the course of scientific medicine during the last half-century, and with the obstacles to the full application of the new knowledge. The writer, naturally, considers more particularly the effects of bacteriology on surgical operations and on the treatment and prevention of infectious diseases. In connection with the latter he finds that the public generally, instead of seeking to secure the application of scientific conclusions which rest upon "as firm a basis of assured knowledge as the phenomena of gravitation," remain passive, or even active, opponents of measures by which many lives might be preserved and much permanent injury to the health of individuals be prevented. Not merely "vestrymen and such-like people," but even some members of Parliament, are included in this condemnation. And this attitude of apathy and resistance is attributed to a want of imaginative capacity to realise either the truths of science or the results which could be secured by their application, and to the absence of the introduction of "physical matters" even into what is supposed to be the highest type of education. It is highly satisfactory that all this should be set forth in the columns of the lay Press, and its exact correspondence with facts is beyond question. Unless public opinion is educated to appreciate the function which science, and science alone, can discharge in practical life, the attainment of the full results of observation and investigation is impossible. Perhaps no illustration of the large scope of this function can be more forcible than the recent work in connection with Malta fever. The discovery that this disease is due to a microbe inhabiting healthy Malta goats and communicated to human beings through their milk, has been followed by certain steps which have led to most impressive results. Hitherto there has been in Haslar Hospital an average of some two hundred patients suffering from this disease, but last month it was reported that not a single case was included among the patients. Here the necessary measures were independent of public support, and if the same thing were true of certain other diseases, parallel action would be followed by equally good effects.

### The General Medical Council and the B.M.A.

The address delivered by the President of the General Medical Council at the opening of the recent session of that body contained an announcement of great interest to the members of the British Medical Association. It appears that the Association has formally decided not only to investigate cases which might require to be brought officially before the Council, but also to act in support of any charges which may seem to be justified. The Association, in short, has determined, as opportunity offers or demands, to act as prosecutor in certain charges which will require the judicial action of the General Medical Council. In these circumstances it is obvious that the position of those members of the Council who are at the same time members of the Association becomes a very delicate one. Acting in the former capacity, they might appear as judges of causes in which, as members of the British Medical Association, they were more or less interested as complainants. The President of the Council has very properly taken legal advice on the matter, and, acting on this advice, has himself resigned his connection with the Association. Further, he has announced that what he has done is, in the opinion of the legal advisers of the Council, imperative on all other members of the Council who happen to be members of the Association, and this, it will be generally allowed, is in accordance with the fitness of things. The judicial actions of the Council must obviously be entirely beyond suspicion of unfairness and partiality, and members of the Council cannot therefore be also members of a prosecuting association. It is a pretty pass to which the present ruling influences have brought the Association. Defeated in their attempt to elect delegates from the Association to be members of the Council, they have broken out in a new place, with the result that no member of the Association can now be considered eligible for a seat on the General Council. In these circumstances it may be questioned whether the Association is fortunate in its "pastors and masters."

